# A molecular survey of febrile cases in malaria-endemic areas along China-Myanmar border in Yunnan province, People’s Republic of China

**DOI:** 10.1051/parasite/2014030

**Published:** 2014-06-23

**Authors:** Xia Zhou, Ji-Lei Huang, Metoh Theresia Njuabe, Sheng-Guo Li, Jun-Hu Chen, Xiao-Nong Zhou

**Affiliations:** 1 Department of Parasitology, Medical College of Soochow University Suzhou 215123 China; 2 National Institute of Parasitic Diseases, Chinese Center for Disease Control and Prevention, WHO Collaborating Centre for Malaria, Schistosomiasis and Filariasis, Key Laboratory of Parasite and Vector Biology Ministry of Health Shanghai 200025 China; 3 Department of Biochemistry, Faculty of Sciences, University of Bamenda North-west Province Cameroon; 4 Tengchong Center for Disease Control and Prevention Tengchong 679100 Yunnan China

**Keywords:** Malaria, Nested PCR, Diagnosis, Myanmar, P.R. China

## Abstract

**Background:** Imported malaria is a major threat to neighboring malaria-eliminating countries such as P.R. China and is difficult to monitor. A molecular survey of febrile patients with a history of traveling abroad along the Myanmar-China endemic border areas from January 2008 to August 2012 was carried out. The rates of infection with species of *Plasmodium* and compliance of microscopy diagnosis with nested PCR (Polymerase Chain Reaction) results were calculated.

**Results:** *Plasmodium* genus-specific nested PCR confirmed that 384 cases were positive. Further species-specific nested PCR showed that the rate of *Plasmodium vivax* infection was 55% (213/384); that of *Plasmodium falciparum* was 21% (81/384) and 17% (67/384) of cases were co-infection cases of *P. vivax* and *P. falciparum*; the remaining 6% (23/384) of cases were caused by other species, such as *Plasmodium ovale*, *P. malaria*, *P. knowlesi* or mixed infections of *Plasmodium*. In total there was 13% (50/384) false microscopy diagnosis including 6% (22/384) error in species diagnosis and 7% (28/384) undiagnosed cases in co-infection or low parasitemia malaria cases.

**Conclusions:** This study indicates that there are considerable numbers of malaria cases in the China-Myanmar endemic border areas that remain undiagnosed or misdiagnosed by microscopy, especially in low-level and/or complex co-infection cases. It is urgent to develop accurate rapid diagnostic tests and apply PCR confirmation for efficient surveillance.

## Abstract (fr)

**Introduction :** Le paludisme importé est une menace majeure pour les pays voisins qui éliminent le paludisme tels que République Populaire de Chine et est difficile à surveiller. Une enquête moléculaire a été réalisée sur des patients fébriles avec une histoire de voyage à l’étranger dans les zones endémiques frontalières Myanmar - Chine de janvier 2008 à août 2012. Les taux d’infection des espèces de *Plasmodium* et de conformité du diagnostic microscopique et des résultats PCR nichée ont été calculés.

**Résultats :** la PCR nichée du genre *Plasmodium* a confirmé que 384 cas étaient positifs au genre. La PCR nichée spécifique aux espèces a montré que le taux d’infection à *P. vivax* était de 55 % (213/384), celui de *P. falciparum* de 21 % (81/384) et que 17 % (67/384) des cas étaient des cas de coinfection de *P. vivax* et *P. falciparum*. Les 6 % restants (23/384) des cas étaient causés par des infections d’autres espèces, telles que *P. ovale*, *P. malariae*, *P. knowlesi* ou des infections de *Plasmodium* mélangées. Au total il y avait 13 % (50/384) de faux diagnostics microscopiques, y compris 6 % (22/384) d’erreurs de diagnostic d’espèce et 7 % (28/384) de diagnostics manquant dans les cas de coinfection ou les cas à parasitémie faible.

**Conclusions :** L’étude indique que des nombres considérables de cas de paludisme dans les zones frontalières endémiques entre Chine et Myanmar restent non diagnostiqués ou mal diagnostiqués par la microscopie, surtout dans le cas de parasitémie faible ou de coinfections complexes. Il est urgent de développer des tests de diagnostic rapide et précis et d’appliquer une confirmation par PCR pour une surveillance efficace.

## Introduction

Malaria is a major infectious disease in the Greater Mekong Subregion (GMS) countries, namely P.R. China, Laos, Myanmar, and Vietnam. In the GMS, Myanmar accounts for approximately one-fifth of the region’s population, more than half of the malaria cases and approximately three-quarters of malaria deaths. “Border malaria” is extremely difficult to monitor; frequent malaria introductions by migratory human populations constitute a major threat to neighboring, malaria-eliminating countries [9]. Yunnan province still has the highest transmission area in P.R. China, particularly in the southern border areas adjacent to Myanmar [7, 8, 30]. In the malaria elimination program, routine surveillance uses the standard method for detection of *Plasmodium* spp. infection by examining Giemsa-stained blood smears under the microscope. Although effective and inexpensive, the method is laborious and time-consuming and its sensitivity drops with the decrease in parasitemia during the disease elimination stage [2]. The Polymerase Chain Reaction (PCR) method has been widely used for the detection and identification of malaria parasites. It was also successfully used to detect parasites in mixed infections, as well as those with low parasitemia [4, 26]. A variety of PCR-based assays have been described for the specific diagnosis of all the five species of *Plasmodium*, i.e. *Plasmodium falciparum, P. vivax*, *P. ovale, P. malariae, and P*. *knowlesi* [23, 25, 32, 22, 5], one of which is based on the sequence of the small subunit ribosomal RNA (ssrRNA) gene used to detect human *Plasmodium* spp. [24].

A molecular survey was carried out of samples collected randomly in the malaria-endemic area along the border line of southern China, in Yunnan province from January 2008 to August 2012. The present study aims to evaluate PCR amplification in comparison with light microscopy examination.

## Materials and methods

### Ethical clearance

The research proposal was approved by the ethics committee at the National Institute of Parasitic Diseases, Chinese Center for Disease Control and Prevention, in particular regarding collecting blood samples from local residents or migrant workers by finger prick. Written informed consent was obtained from all adult participants and from the parents, or legal guardians of children. Any participant found to be parasitologically positive received anti-malaria treatment, except for those with contraindications.

### Patient samples and microscopy examination

The population of the Tengchong village is served by a general hospital, a polyclinic at the village’s Center for Disease Control and Prevention (CDC), and 15 government health clinics. A total of 562 blood specimens were collected from febrile patients in China-Myanmar endemic border areas at Tengchong Hospital, the polyclinic of the village’s CDC and the government health clinics. All these patients in Tengchong village come from different countries and most of (82.6%, 464/562) these febrile patients had a history of being abroad in Myanmar during the last year. Finger-prick blood samples were collected and the Giemsa-stained blood smears were examined by microscope from January 2008 to August 2012. Thick blood smears were examined under 1000× magnification by microscopists with extensive experience in identification of malaria parasites. The thick film was considered positive when malaria parasites were present and negative if no parasites were seen after 500 leukocytes were counted. In total, 373 samples including 214 *P. vivax* infection cases, 96 *P. falciparum* cases and 63 cases of vivax and falciparum co-infection were referred to as the Microscopy-Positive group (MP group). The remaining 189 samples were the Microscopy-Negative group (MN group). Parasite density per 200 leukocytes was counted, then calculated as the number of parasites per microliter by assuming a leukocyte count of 7000.

### DNA extraction

DNA was extracted from the blood spots using the QIAamp DNA Mini Kit (QIAGEN China (Shanghai) Co., Ltd.) according to the manufacturer’s instructions and the method of Foley et al. [12]. Approximately 30 μL of blood were used per filter spot. The final template volume obtained after Chelex extraction was 150 μL. Throughout the process, negative controls were included to ensure lack of contamination.

### PCR amplification

Nested PCR amplification was performed according to Snounou et al. [24] using a Biometra Thermocycler (England). For the first PCR, 1 μL of template DNA was added to a 20-μL PCR mixture consisting of 0.4 M of each primer (rPLU1 and rPLU5), 10 μL 2× Taq PCR MasterMix (Tiangen Biotech, Beijing, China) containing 0.1 U Taq polymerase/μL, 500 μM deoxynucleotide triphosphates (dNTP), 3 mM MgCl_2_, 100 mM KCl, 20 mM Tris-HCl, pH 8.3. DNA amplification was carried out under the following conditions: 94 °C for 5 min, 30 cycles of (94 °C for 30 s, 55 °C for 30 s, and 72 °C for 1 min), followed by a final extension at 72 °C for 5 min. One microliter of the first PCR product was used in the second amplification. Conditions and concentrations used for the second amplification were identical to those used for the first, except that rPLU3/rPLU4 were used as primers (Table 1) and amplification was performed over 35 cycles. The size of the DNA target, amplified by these outer primers, is about 1600–1700 bp and by the inner primers is 235 bp. These primers are genus-specific, and can therefore amplify the target sequences from all five species of the human malaria parasite, *P. falciparum, P. vivax, P. ovale, P. malariae and P*. *knowlesi*. Detection of *Plasmodium* species was performed using five species-specific primer pairs. Because of the primer pair Pmk8 and Pmkr9 may cross-hybridize with the corresponding sequence of *P. vivax* because of the high similarity of 18S SSU rRNA gene sequences among these parasites [13]. Knowlesi malaria infection cases were confirmed by a new set of primers (Pkr140-5F, Pkr140-5R) with application in a Single-Step PCR Assay for the detection of *P. knowlesi* [18]; all the primers were also applied: see Table 1. If the genus-specific nested PCR results were positive, these first PCR products were used as the template in the next species nested PCR amplification under the same conditions. The negative control reaction was established in each amplification reaction. The positive controls of *P. falciparum* and *P. vivax* were, respectively, set by the 3D7 strain of *P. falciparum* and isolate of *P. vivax* from a malaria patient of Yunnan province preserved by our laboratory. Electrophoresis of the PCR products was performed using 2% agarose gel followed by Gold-View staining and visualizing under a UV light. All the positive results of infected *P. ovale, P*. *knowlesi* and *P. malariae* infection cases and several *P. vivax* and *P. falciparum* ones plus all the co-infection cases were again confirmed by sequencing of PCR products by BGI Company, China. Sequences obtained by direct sequencing of nested-PCR products of second rounds from these co-infection cases above were aligned by using the ClustalW method (EMBL-EBI, Hixton, and Cambridge, UK) and analyzed by MEGA version 5.1 (http://mega.software.informer.com/5.1b/)

**Table 1.** Primer sequences for PCR detection of five human malaria parasites

	*Plasmodium* spp	Size (bp)	Primers	Primer sequences (5′→3′)
Nested 1st round primers	Genus-specific	1600–1700	rPLU1	TCAAAGATTAAGCCATGCAAGTGA
			rPLU5	CCTGTTGTTGCCTTAAACTTC
Nested 2nd round primers	Genus-specific	235	rPLU3	TTTTTATAAGGATAACTACGGAAAAGCTGT
			rPLU4	TACCCGTCATAGCCATGTTAGGCCAATACC
Nested 2nd round species-specific primers	*P. vivax*	121	rVIV1	CGCTTCTAGCTTAATCCACATAACTGATAC
			rVIV2	ACTTCCAAGCCGAAGCAAAGAAAGTCCTTA
	*P. falciparum*	206	rFAL1	TTAAACTGGTTTGGGAAAACCAAATATATT
			rFAL2	ACACAATGAACTCAATCATGACTACCCGTC
	*P. ovale*	226	rOVA1	ATCTCTTTTGCTATTTTTTAGTATTGGAGA
			rPLU2	ATCTAAGAATTTCACCTCTGACATCTG
	*P. malariae*	145	rMAL1	ATAACATAGTTGTACGTTAAGAATAACCCC
			rMAL2	AAAATTCCCATGCATAAAAATTATACAAA
	*P. knowlesi*	153	Pmk8	GTTAGCGAGAGCCACAAAAAAGCGAAT
			Pmkr9	ACTCAAAGTAACAAAATCTTCCGTA
Single-Step PCR primers	*P. knowlesi*	200	Pkr140-5F	CAGAGATCCGTTCTCATGATTTCCATGG
			Pkr140-5R	CTRAACACCTCATGTCGTGGTAG

## Results and discussions

**Figure 1.**
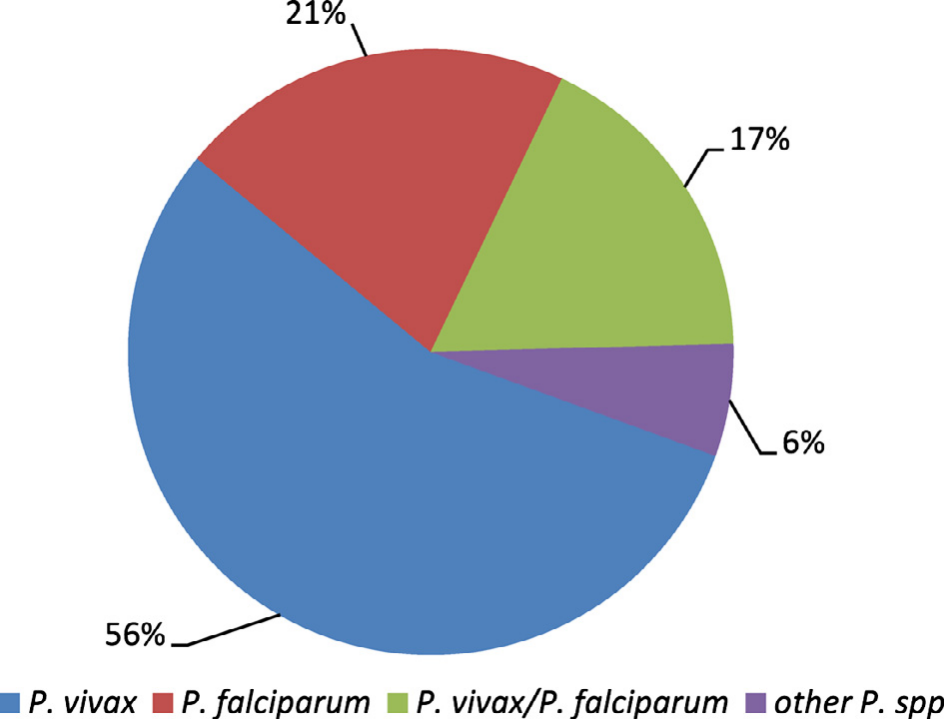
*Plasmodium* species identified in 384 malaria patients from China-Myanmar endemic areas form Yunnan province, January 2008 to August 2012.

**Figure 2.**
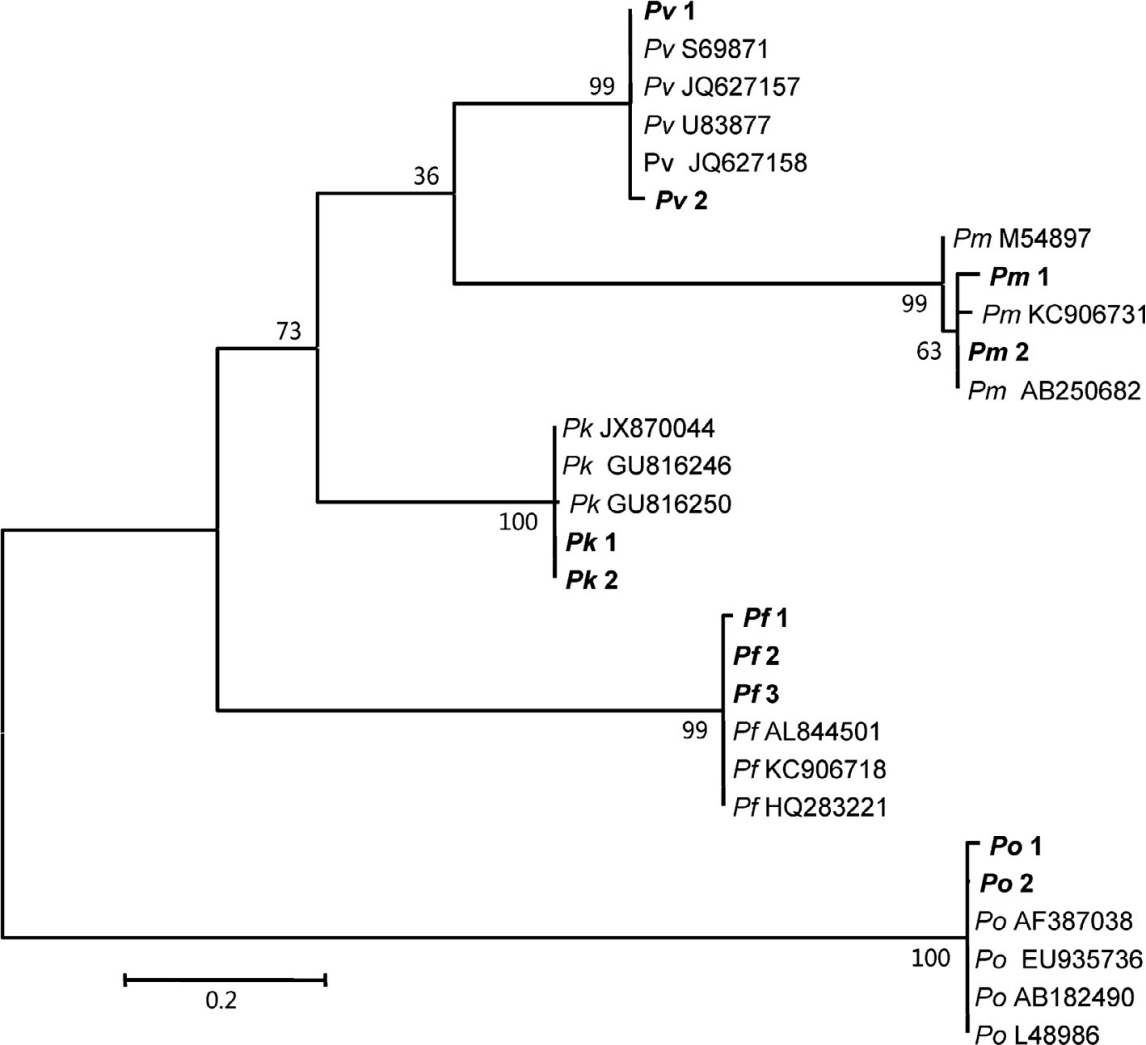
Phylogenetic trees based on the partial sequences of 18S ribosomal RNA gene of five *Plasmodium* from malaria patients in China-Myanmar endemic areas from Yunnan province, January 2008 to August 2012. Phylogenetic analysis produced by the neighbor-joining method using MEGA version 5.1 software. Partial sequences of 18S ribosomal RNA of our case study samples are denoted as Pv1, Pv2, Pm1, Pm2, Pk1, Pk2, Pf1, Pf2,Pf3, Po1, and Po2 in bold face with other *Plasmodium* species. Scale bar indicates nucleotide substitutions per site.

All 562 specimens were collected and the Giemsa-stained blood smears were examined under the microscope. In total, 373 cases were positive by microscopy (MP group), while the remaining 189 samples were diagnosed as negative (MN group). *Plasmodium* genus-specific nested PCR confirmed that 384 cases were positive (371 cases from the MP group with 214 cases of *P. vivax* infections, 96 cases of *P. falciparum* infections and 61 cases of co-infections of *P. vivax and P. falciparum*, and 13 from the MN group of 189 febrile patients). So, the nested PCR confirmed all of the MP group malaria cases (100%, 371/371) and diagnosed 13 malaria cases in the MN group (6.9%, 13/189) which may have been ignored because of the low parasitemia conditions. Further species-specific nested PCR identified that among the 384 malaria cases, the *P. vivax* infection rate was 55.5% (213/384), that of *P. falciparum* was 21.1% (81/384) and 17.4% (67/384) of cases were co-infections of *P. vivax* and *P. falciparum*, with 6.0% (23/384) of cases caused by other infections, including five cases of *P. ovale* and three of *P. malariae*; and various cases of mixed infections: two cases of *P. knowlesi* and *P. falciparum*, three cases of *P*. *vivax*, *P*. *falciparum* and *P. ovale*, two cases of *P*. *vivax* and *P. ovale*, two cases of *P*. *falciparum* and *P. ovale*, two cases of *P*. *falciparum* and *P. malariae*, one case of *P*. *vivax, P*. *falciparum* and *P. ovale*, one case of *P*. *vivax*, *P. malariae* and *P. ovale*, one case of *P*. *vivax*, *P*. *falciparum*, *P. malariae* and *P. ovale*, and one case of *P. malariae* and *P. ovale*. In total there was 13.0% (50/384) error in microscopy diagnosis including 5.7% (22/384) error in species diagnosis and 7.3% (28/384) undiagnosed cases in co-infection or low parasitemia malaria cases (see the details of all the species of 384 malaria infection cases in Table 2 and Fig. 1). Phylogenetic trees were produced by using the neighbor-joining method in MEGA version 5.1 The case samples (denoted as Pv1, Pv2, Pm1, Pm2, Pk1, Pk2, Pf1, Pf2, Pf3, Po1, and Po2) clustered with other *Plasmodium* isolates (see Fig. 2).

**Table 2.** *Plasmodium* species identified in 384 malaria patients in China-Myanmar endemic border areas in Yunnan province, January 2008 to August 2012

Species-specific PCR results	Microscopy positive (371)			Microscopy negative	Total no. (%)
	*P. vivax*	*P. falciparum*	*P. vivax / P. falciparum*		
	214	96	61	189	
*P. vivax*	195	6	0	12	213 (55.5)
*P. falciparum*	6	74	0	1	81 (21.1)
*P. vivax/P. falciparum*	3	5	59	0	67 (17.4)
*P. ovale*	2	2	1	0	5 (1.3)
*P. malariae*	2	0	1	0	3 (0.8)
*P. falciparum/P. vivax* **/** *P. ovale*	1	2	0	0	3 (0.8)
*P. vivax* **/** *P. ovale*	2	0	0	0	2 (0.5)
*P. falciparum* **/** *P*. *knowlesi*	0	2	0	0	2 (0.5)
*P. falciparum* **/** *P. malariae*	0	2	0	0	2 (0.5)
*P. falciparum* **/** *P. ovale*	1	1	0	0	2 (0.5)
*P. falciparum/P. vivax* **/** *P. malariae*	1	0	0	0	1 (0.3)
*P. ovale* **/** *P. malariae*	1	0	0	0	1 (0.3)
*P. vivax* **/** *P. ovale* **/** *P. malariae*	1	0	0	0	1 (0.3)
*P. falciparum/P. vivax* **/** *P. ovale* **/** *P. malariae*	0	1	0	0	1 (0.3)

The long border of 4061 km in southern Yunnan, directly adjacent to the northwest of Laos, northeast of Myanmar and north of Vietnam, is considered the most prevalent area of malaria in P.R. China [8]. Despite the reduction of malaria over the last decade in China, care must be taken to prevent any new foci of epidemics, after launching the National Malaria Eradication Program (NMEP) as a consequence of different factors. Control and eventual elimination of human parasitic diseases in P.R. China requires novel, innovative approaches, particularly in areas of diagnostics, mathematical modeling, monitoring, evaluation, surveillance and public health response [6]. In addition, the movement of sections of the population that includes tourism, border trade business, traveling and holidays, particularly in those free ports on the border, may affect the mode of severity and endemics of malaria in this region [8].

Low-level infection appears to be common across malaria-endemic areas, often as complex mixed infections. A number of studies demonstrated that PCR is more reliable than microscopy in detection of malaria in low parasitemic areas [14, 20, 21]. Several malaria infections from endemic countries are reported to be sub-patent, with very low parasitemia [28]. Due to false negative diagnosis by microscopy in endemic areas, a number of patients remain untreated and they may play a role as carriers of malaria parasites. Microscopy analysis is currently used for identification of *Plasmodium* species, a technique appropriate for routine clinical diagnosis. However, its sensitivity is limited and diagnostic accuracy depends greatly on the particular expertise of laboratory staff. The detection limit of malaria parasites by microscopy is 20–30 parasites/μL of blood [17]. Frequently, however, due to specimen load pressure, inadequate staining and poorly maintained microscopes, even this limit may be decreased in both quality and quantity. The PCR method not only detected almost all of the microscopy-positive samples, but also detected mixed infections and malaria cases that were not diagnosed by microscope [4, 14].

It is reported that *P. ovale*, rarely seen in P.R. China, was distributed widely in Myanmar [29]; here, we detected more than 3% ovale malaria cases in China-Myanmar endemic border areas. Our species detection work also improved as Kawamoto et al. [16] reported that there might be a higher prevalence of *P. ovale*, and *P. malariae* in East Asia than previously reported when using alternative diagnostic methods, such as acridine orange staining and PCR-based methods. The rate of *P. knowlesi* co-infections with other malaria parasites was reported to be about 30% in southern Myanmar near Yunnan Province of China [15]. The *P. knowlesi* co-infection rate detected in this study was much lower than that reported in southern Myanmar, which may be because all the field samples we collected were mainly in the villages adjacent to northeastern Myanmar, so only two *knowlesi* malaria cases were detected. To establish long-term cooperation in prevention and control of important infectious diseases in cross-border areas, a proposal for malaria prevention and control in border areas of Yunnan has been suggested. A more sensitive and specific diagnosis method is needed to obtain a rapid-response emergency mechanism for the important infectious diseases [1].

Artemisinin resistance in *P. falciparum* malaria has already been reported [10, 19]. Uhlemann and Fidock [27] reported that *Plasmodium* loss of malarial susceptibility to artemisinin in Thailand suggested that the resistance gene also exists in *P. vivax*-endemic areas. The latest research work in northern Angola has demonstrated that because *P. malariae* and *P. ovale* are sympatric with *P. falciparum* across the continent and are frequently present as co-infections, populations of *P. falciparum* previously dominated by chloroquine (CQ)-resistant genotypes are now under different drug selection pressures. In short, it is essential that the treatment of malaria patients depends on the correct diagnosis of malaria species, particularly with the spread of parasitic resistance to antimalarial drugs [3, 11]. Thus, controlling malaria may face long-term difficulties.

Our latest study showed that human babesiosis caused by another intraerythrocytic protozoan, *Babesia microti*, is emerging in China-Myanmar border areas in Yunnan province, P.R. China [31]. So, the co-prevalence of *Babesia* in malaria-endemic regions is possible and sometimes babesiosis has been diagnosed as malaria due to the similarities of symptoms and morphology. Laboratories in babesiosis-endemic areas need broad access to modern diagnostic methods for a more rapid and reliable microbiological diagnosis in cases of suspected cases of malaria and/or human babesiosis. We should also pay attention to *P. ovale*, *P. knowlesi* and *P. malariae* as well as *Babesia*, for there may be co-infection with the other two dominant *Plasmodium* species.

## Conclusion

This study indicates that there are considerable numbers of malaria cases in the China-Myanmar endemic border areas that remain undiagnosed or misdiagnosed by microscopy, especially in low-level and/or complex co-infection cases. It is urgent to develop accurate, rapid diagnostic tests suitable for these intraerythrocytic protozoans besides the two dominant plasmodium species, and apply PCR confirmation for accurate surveillance.
